# Risk Factors of CVD Mortality among the Elderly in Beijing, 1992 – 2009: An 18-year Cohort Study

**DOI:** 10.3390/ijerph110202193

**Published:** 2014-02-21

**Authors:** Tao Zhou, Xia Li, Zhe Tang, Changchun Xie, Lixin Tao, Lei Pan, Da Huo, Fei Sun, Yanxia Luo, Wei Wang, Aoshuang Yan, Xiuhua Guo

**Affiliations:** 1 School of Public Health, Capital Medical University, 10 Xitoutiao, Youanmen, Beijing 100069, China; E-Mails: zhoutaosmile@126.com (T.Z.); lixia_new@163.com (X.L.); taolixin.2008@163.com (L.T.); panlei_helen@163.com (L.P.); lyx100@ccmu.edu.cn (Y.L.);; 2 Beijing Municipal Key Laboratory of Clinical Epidemiology, 10 Xitoutiao, Youanmen, Beijing 100069, China; 3 Epidemiology and Public Health, University College Cork, Western Road, Cork 78746, Ireland; 4 Xuan Wu Hospital, Capital Medical University, 45 Changchun Street, Beijing 100069, China; E-Mails: tangzhe@medmail.com.cn (Z.T.); sf69@163.com (F.S.); 5 Division of Epidemiology and Biostatistics, Department of Environmental Health, University of Cincinnati, Ohio, OH 45267, USA; E-Mail: xiecn@ucmail.uc.edu; 6 Institute for Infectious Disease and Endemic Disease Control, Beijing Center for Disease Prevention and Control, No. 16 Hepingli Middle Street, Dongcheng District, Beijing 100013, China; E-Mail: howardhuo@139.com; 7 School of Medical Science, Edith Cowan University, 2 Bradford Street, Mount Lawley, Massachusetts, WA 6050, Australia; E-Mail: wei6014@yahoo.com; 8 Beijing Municipal Science and Technology Commission, Sijiqing Street, Beijing 100195, China

**Keywords:** cardiovascular disease, competing risk model, Fine and Gray’s test, risk factors

## Abstract

Few researchers have examined the effects of multiple risk factors of cardiovascular disease (CVD) mortality simultaneously. This study was to determine the associations of combined lifestyle and other factors with CVD mortality among the elderly (n = 3,257), in Beijing, China, through data mining of the Beijing Longitudinal Study of Aging (BLSA). BLSA is a representative cohort study from 1992 to 2009, hosted by Xuan Wu Hospital. Competing risk survival analysis was conducted to explore the association between risk factors and CVD mortality. The factors focused mainly on lifestyle, physical condition, and the model was adjusted for age and gender. There were 273 of the 1,068 recorded deaths caused by CVD among the 2010 participants. Living in a suburban area (HR = 0.614, 95% CI: 0.410-0.921) was associated with lower CVD mortality. Increasing age (66–75: HR = 1.511, 95% CI: 1.111–2.055; ≥76: HR = 1.847, 95% CI: 1.256–2.717), high blood pressure (HR = 1.407, 95% CI: 1.031–1.920), frequent consumption of meat (HR = 1.559, 95% CI: 1.079–2.254) and physical inactivity (*p =* 0.046) were associated with higher CVD mortality. The study provides an instructional foundation for the control and prevention of CVD in Beijing, China.

## 1. Introduction

Cardiovascular disease (CVD) is an important worldwide public-health challenge because of its high prevalence and mortality [1,2,3]. It accounted for 30% of all global deaths in 2011, according to the World Health Organization [4,5] and CVD has also become the top cause of diseases and deaths among the Chinese [6]. As the greater likelihood of early death and huge disability-adjusted life-years caused by CVD, the discussion of the risk factors for CVD would allow public-health policy-makers to assign effective priority and resources to its management and prevention [7]. 

The increasing prevalence of CVD results from the aging population, and Beijing has already entered the aged society status in 1990 [8,9]. Many epidemiologic studies have examined the association of individual lifestyle practices, such as smoking, physical activities, *etc*. [10,11], but few studies have addressed the effects of simultaneous multiple risks factors as well as considering the competing risk events. 

Survival techniques are well developed and implemented in major statistical software to the time-to-event analysis. Yet, there are some situations where it may not be appropriate to apply the usual survival methods. Such as analysis of the data when competing risk are present requires specific methods, the Kaplan-Meier method has been shown to overestimate the probability of death in comparison with the more specific competing risk model [12,13]. Individuals are generally observed from the study entry until the occurrence of the event of interest, a competing risk event, or censoring [14,15,16]. In general, a competing risks situation arises when an individual can experience more than one type of event and the occurrence of one type of event hinders the occurrence of other types of events. [17,18]. The aims of the present study were to determine the major predictors of CVD mortality by a comprehensive model, while incorporating the competing events of the alternate outcomes into the analysis.

## 2. Methods

### 2.1. Study Design and Population

Data were derived from the BLSA, a community-based prospective cohort study hosted by Xuan Wu Hospital in Beijing, which began in August 1992. A random sample of 3,257 community dwellers aged 55 years or above was recruited from three districts: Xuanwu (urban), Daxing (suburban), Huairou (rural). All participants completed a questionnaire regarding lifestyle and health behaviors, *ets.*, and 2,101 subjects who had undergone routine blood biochemical examination at baseline were selected from the 3,257 participants. Because of missing data, 2,010 subjects were finally included in the analysis.

### 2.2. Assessment of Risk Factors

The study was mainly focused on lifestyle, physical condition and dietary habits. Several conditions (high blood pressure, high blood glucose, and blood fat) which could be consequences of an unhealthy lifestyle or dietary habits were also included. Activities of Daily Living (ADL) scale and Center for Epidemiological Studies Depression (CES-D) scale were detailed measured. All risk factors were assessed by self-reporting on the questionnaires with a high degree of reliability and accuracy. The follow-up studies were conducted every two or three years. Baseline values were used in this analysis to minimize the potential of clinical or subclinical diseases affecting the risk factor status.

Age was divided into several groups according to the initial age. Height, BMI (Body Mass Index) [19], blood pressure (BP), smoking status, drinking status, and the frequency of physical activities were measured and assessed at the beginning of the study. Blood samples were collected after an overnight fasting of at least 12 h. Glucose, high density lipoprotein (HDL), and triglycerides (TG) were subsequently measured. BP was measured on the right arm of seated subjects and at rest at least 10 min by a trained nurse. Then the subjects were divided into normal or abnormal groups according to the standard of diabetes mellitus [20] and dyslipidemia [21]. Dietary factors were assessed by a food frequency questionnaire. The average consumption of fish, meat, vegetables, fruit, and quantity of salt were reported by the subjects. According to the results of Latent Class Model [22], the subjects were divided into three groups. The first group represented sufficient nutrients, the second group comprised the intermediate-type diets, and the third group consumed meat-based diets. Generally, the intake of milk, fruits, bean products, and eggs were lower in the second and the third group. Physical limitation was assessed using the 12 items of ADL, which consists of two categories: Instrumental Activities of Daily Living (IADL) and Basic Activities of Daily living (BADL) [23]. The elderly were divided into self-care ability and self-care disability. In this study, CESD was used to assess depression symptoms. The total score was 60 and the standard cut-off value was 16 [24]. 

### 2.3. Ascertainment of Mortality

The main outcome was death from all causes, occurring after the return of 1992 questionnaire but before 31 December 2009. Years of follow-up were accrued from the return data of the questionnaire until either of the following first occurs: death, loss to follow-up or the end of follow-up. Causes of death were grouped into four broad categories indicated by the International Classification of Disease, ninth revision (ICD-9) or ICD-10: Cardiovascular disease, cerebrovascular disease (CBVD), cancer and other diseases. The primary interested endpoint was death from CVD. Other causes of death comprised the competing risk events.

### 2.4. Statistical Analysis

Multiple Imputation (MI) was performed to fill in a few missing serum biochemical data. The Markov chain Monte Carlo method was chosen to avoid the loss of generality, which have considered the distribution of data. The MI procedure of SAS software package (version 9.2; SAS Institute, Chicago, IL, USA) was used [25]. 

The competing risk model was extended from Cox’s proportional hazards model to competing-risks data and it has considered the sub-distribution hazard [26,27]. Contrary to a cause-specific analysis that censor competing event(s), the Fine-Gray approach “carries forward” the competing event(s) in the risk set with appropriate weighting [28,29,30,31,32,33,34,35,36,37,38,39]. In addition, the cumulative incidence functions (CIF) between different groups were compared, such as gender, status of self-health assessment, and so on. Univariate and multivariate analysis were used to identify the association between covariates and CVD mortality. Statistical significance was considered as a 2-sided p-value of less than 0.05. The results of univariate analysis (*p* value < 0.3) were the criteria for inclusion of risk factors in the final multivariate model. Basic statistical analysis was performed by SAS version 9.2. Competing risk analysis was implemented in R (version 3.0.2) [30,31].

## 3. Results

### 3.1. Basic Characteristics and the CIF of Death

**Figure 1:**
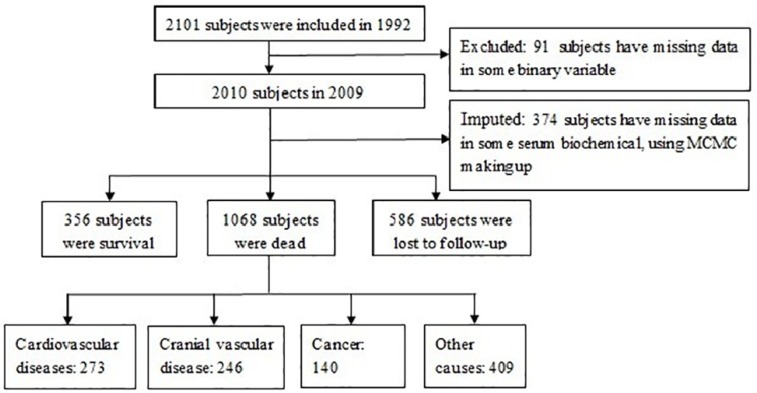
The population flow chart.

**Figure 2:**
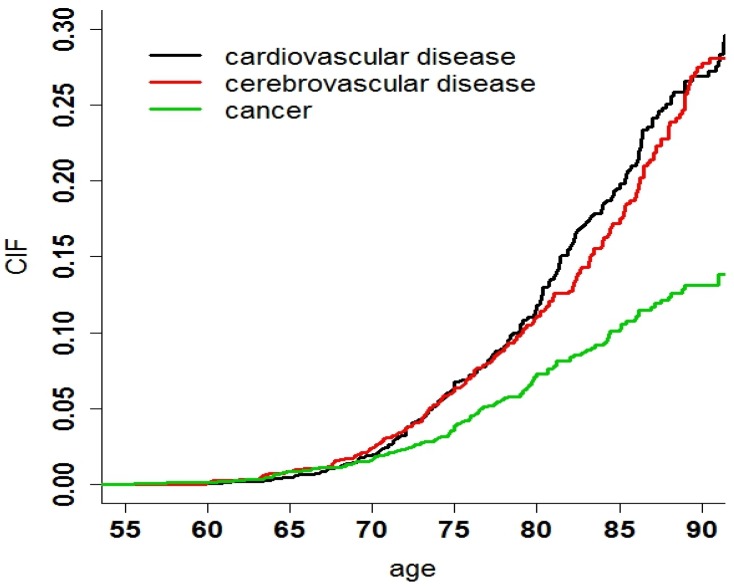
CIFs for three main outcomes: CVD, CBVD and cancer.

Note: Abbreviations: CVD = cardiovascular disease; CBVD = cerebral vascular disease.

A total of 2,010 participants were included in the analysis. The number of participants who were excluded and the reasons for their exclusion are shown in Figure 1. The enrolled and the missed subjects were compared to assess enrolment bias, the differences of characteristics between these two groups were not statistical significant (*p* < 0.05). By the end of follow-up in 2009, there were 356 surviving subjects, 585 missing subjects, and 1,068 deaths. Among the 1,068 deaths, 273 were caused by CVD (25.54%), 246 by cerebrovascular disease (23.01%), 140 by cancer (13.10%), and 409 were caused by “other causes” (38.35%), shown in Table 1. At the end of follow-up, considering the competing risks, the CIF of CVD death was 0.19, CBVD was 0.17, and cancer was 0.10. Additionally, the age of death was used as the abscissa to adjust the different distribution of age in different groups. The CIF of death due to CVD at age 85 was 0.20, cerebrovascular disease was 0.16, and cancer was 0.11(Figure 2).

**Table 1:** Characteristics of subjects in Beijing between 1992 and 2009.

Characteristic		Total Subjects (%)	Total Deaths (%)	CVD Deaths (%)
Total		2,010 (100)	1,068 (100)	273 (100)
Gender	male	987 (49.104)	545 (51.030)	133 (48.718)
	female	1,023 (50.509)	523 (48.970)	140 (51.282)
Age group	55–65	705 (35.075)	246(23.034)	67 (24.542)
	66–75	728 (36.219)	408(38.202)	106 (38.828)
	≥76	577 (28.706)	414 (38.764)	100 (36.630)
Smoke	no	593 (29.502)	738 (69.101)	83 (30.403)
	yes	1,417 (70.498)	330 ( 30.899)	190 (69.597)
Drink	no	1,569 (78.060)	832 (42.24)	222 (81.319)
	yes	441 (21.940)	236 (22.097)	51 (18.681)
Depression	no	1,649 (82.040)	849 (79.494)	215 (78.755)
	yes	361 (17.960)	219 (20.506)	58 (21.245)
Sad event	no	1,364 (67.861)	724 (67.790)	177 (64.835)
	yes	646 (32.139)	344 (32.210)	96 (35.165)
Exercise	no	806 (40.100)	419 (39.232)	111 (40.659)
	yes	1,204 (59.900)	649 (60.768)	162 (59.341)
BADL	normal	1,936 (74.378)	1,010 (94.570)	264 (96.703)
	disability	74 (25.622)	58 (5.431)	9 (3.297)
IADL	normal	1,495 (74.378)	696 (65.169)	183 (67.033)
	disability	515 (25.622)	372 (34.831)	90 (32.967)
Marital	have a spouse	1,354 (67.363)	645 (60.393)	135 (49.451)
	mateless	656 (32.637)	423 (39.607)	90 (32.967)
Self-report	health	1,639 (81.542)	826 (77.341)	211 (77.289)
	not health	371 (18.458)	242 (22.659)	62 (22.711)
Diabetes	abnormal	1,698 (84.478)	876 (82.022)	222 (80.889)
	normal	312 (15.522)	192 (17.978)	51 (19.111)
Blood lipid	abnormal	500 (24.876)	251 (23.502)	65 (24.4)
	normal	1,510 (75.124)	817 (76.498)	208 (75.6)
BP	Sbp ≤ 120 or dbp ≤ 80	551 (27.413)	245 (22.940)	60 (21.978)
	Sbp > 120 or dbp > 80	255 (12.687)	106 (9.925)	22 (8.059)
	Sbp ≥ 140 or dbp ≥ 90	1,240 (61.692)	717 (67.135)	191 (69.963)
Education	college or above	137 (6.816)	48 (4.494)	13 (4.762)
	high school	96 (4.776)	34 (3.184)	14 (5.128)
	junior diploma	165 (8.209)	73 (6.835)	18 (6.593)
	primary school	579 (28.806)	283 (26.498)	64 (23.443)
	illiterate	1,033 (51.393)	630 (58.989)	164 (64.073)
BMI	normal	1,102 (54.826)	579 (54.213)	155 (56.777)
	thin	376 (18.706)	239 (22.378)	58 (21.245)
	overweight	276 (13.731)	118 (11.049)	26 (9.524)
	obesity	256 (12.736)	132 (12.360)	34 (12.454)
Residence	rural	283 (14.080)	168 (15.730)	50 (18.315)
	suburban	508 (25.274)	338 (31.648)	58 (21.245)
	urban	1,219 (60.647)	562 (52.622)	165 (60.440)
Diet	intermediate-type diet	1,016 (50.547)	569 (53.277)	131 (47.985)
	sufficient nutrients	693 (34.478)	300 (28.090)	85 (31.136)
	meat based diet	301 (14.975)	199 (18.633)	57 (20.879)

### 3.2. Competing Risk Model

Table 2 shows the association of each risk factor with CVD mortality. After considering competing risks of death, the mortality rates of the elderly without spouse, disabilities assessed by IADL, and poor self-assessed health were respectively at a higher risk than those who had a spouse, able-bodied, and with a healthy self-assessment. Additionally, subjects aged above 76, with high blood pressure, consuming more meat and illiterate were also associated with higher CVD mortality risk. Overweight, living in suburban, consuming sufficient nutrient were associated with a lower CVD mortality. In the final model, after all of the adjustments, the risk of CVD mortality increased sharply with age (66 ≤ age ≤ 75: HR = 1.511, 95% CI: 1.111–2.055, age ≥ 76: HR = 1.847, 95% CI: 1.256–2.717). Subjects with hypertension were at a higher risk of CVD death (HR = 1.407, 95% CI: 1.031–1.920). And the CVD mortality of the elderly in suburban was significantly lower than that of the elderly in the rural area (HR = 0.614, 95% CI: 0.410–0.921). In addition, frequent consumption of meat was associated with increased risk of CVD mortality (HR = 1.518, 95% CI: 1.044–2.207) (Table 2). 

Besides, the same analysis was subsequently repeated after further stratification according to gender. Univariate analysis for male showed height was inversely related to mortality of CVD. Disability assessed by IADL, excessive drinking, without spouse, poor self-health rated, age above 76, with hypertension, illiterate and consuming more meat were positively associated with increased risk of CVD mortality. Multivariate analysis showed age, BMI and diet were associated with CVD mortality (Table 3). Univariate analysis for female showed age and hypertension were associated with rising CVD mortality, multivariate analysis also showed consuming more meat significantly increased CVD mortality (Table 4). Additionally, no significant interactions were demonstrated.

**Table 2:** Predictors of CVD mortality, using competing risks models.

**Variables**	**Univariate Analysis**			**Multivariate Analysis**		
	HR	95%CI	*p* value	HR	95%CI	*p* value
Gender (male)	0.530	0.732–1.171	0.530	-	-	-
Height	0.989	0.976–1.011	0.120	1.006	0.989–1.023	0.491
Depression (normal)	0.977	0.756–1.262	0.150	1.102	0.800–1.518	0.546
Smoking (no-smoke)	0.908	0.966–1.887	0.861	-	-	-
Drink (no-drink)	1.270	0.942–1.723	0.122	1.215	0.876–1.686	0.238
Sad-event (experienced)	0.887	0.693–1.141	0.341	-	-	-
Exercise (always)	0.996	0.783–1.274	0.983	-	-	-
BADL (can take care)	0.915	0.465–1.798	0.824	-	-	-
IADL (can take care)	1.540	1.231–1.990	<0.001	1.172	0.860–1.596	0.320
Marital status ( have a spouse)	1.370	1.073–1.754	0.011	0.998	0.730–1.336	0.939
Assessment of health (normal)	1.340	1.011–1.790	0.043	1.141	0.831–1.568	0.410
Blood lipid (normal)	0.932	0.707–1.232	0.621	-	-	-
Diabetes (normal)	1.290	0.954–1.741	0.100	1.332	0.972–1.823	0.074
Age-group (55–65)						
66–75	1.122	0.883–1.431	0.341	1.511	1.111–2.055	0.008
≥76	1.692	1.321–2.164	<0.001	1.847	1.256–2.717	0.002
Blood-pressure (normal)						
Sbp > 120 or dbp > 80	0.577	0.374–0.889	0.013	0.799	0.488–1.308	0.370
Sbp ≥ 140 or dbp ≥ 90	1.620	1.260–2.133	<0.001	1.407	1.031–1.920	0.032
Education level (graduate)						
high school diploma	1.150	0.675–1.960	0.610	1.519	0.715–3.228	0.280
Junior diploma	0.748	0.468–1.191	0.220	1.102	0.535–2.267	0.769
primary school	0.745	0.564–0.984	0.038	1.129	0.603–2.113	0.701
Illiterate	1.450	1.141–1.840	<0.001	1.525	0.815–2.853	0.189
Body-mass-index (normal)						
thin	1.180	0.888–1.580	0.250	1.074	0.783–1.471	0.660
overweight	0.630	0.421–0.942	0.025	0.673	0.439–1.031	0.069
obesity	0.969	0.678–1.390	0.870	0.965	0.652–1.428	0.861
Area (rural)						
suburban	0.728	0.544–0.974	0.033	0.614	0.410–0.921	0.018
urban	1.080	0.851–1.379	0.520	1.080	0.707–1.651	0.720
Diet (intermediate-type diets)						
sufficient nutrients	0.852	0.662–1.104	0.210	0.954	0.694–1.312	0.768
meat based diets	1.460	1.093–1.949	0.012	1.559	1.079–2.254	0.018

**Table 3:** Predictors of CVD mortality in male, using competing risks models.

**Variables**	**Univariate analysis**			**Multivariate analysis**			
	HR	95%CI	*p* value	HR		95%CI	*p* value
Height	0.968	0.945–0.991	0.008	0.984		0.954–1.012	0.281
Depression (normal)	1.129	0.715–1.812	0.590	–		–	–
Smoking (no smoke)	1.110	0.795–1.560	0.544	–		–	–
Drink (no drink)	1.431	1.100–2.041	0.0480	1.375		0.955–1.980	0.087
Sad event (experienced)	1.051	0.734–1.532	0.794	–		–	–
Exercise (always)	1.157	0.824–1.689	0.413	–		–	–
BADL (can take care)	0.773	0.243–2.445	0.662	–		–	–
IADL (can take care)	1.909	1.320–2.769	<0.001	1.462		0.955–2.238	0.081
Marital status (have a spouse)	1.469	1.020–2.131	0.044	1.125		0.729–1.741	0.586
Assessment of health (normal)	1.550	1.020–2.351	0.041	1.076		0.679–1.714	0.746
Blood lipid (normal)	1.211	0.795–1.801	0.386	–		–	–
Diabetes (normal)	1.220	0.789–1.889	0.371	–		–	–
Age-group (55–65)							
66–75	1.120	0.794–1.567	0.535	1.622		1.021–2.582	0.041
≥76	1.671	1.189–2.345	0.003	1.681		0.965–2.930	0.046
Blood pressure(normal)							
Sbp > 120 or dbp > 80	0.536	0.289–0.992	0.047	0.817		0.406–1.64	0.570
Sbp ≥ 140 or dbp ≥ 90	1.751	1.220–2.510	0.002	1.362		0.880–2.101	0.168
Body mass index (normal)							
thin	1.110	0.721–1.697	0.641	1.237		0.763–2.013	0.389
overweight	0.604	0.336–1.089	0.092	0.769		0.404–1.459	0.418
obesity	1.712	1.121–2.620	0.013	1.889		1.159–3.078	0.011
Area (rural)							
suburban	0.696	0.457–1.058	0.092	0.631		0.342–1.164	0.140
urban	1.093	0.774–1.529	0.630	1.154		0.599–2.220	0.671
Diet (intermediate-type diets)							
sufficient nutrients	0.768	0.536–1.089	0.150	0.806		0.513–1.272	0.350
meat based diets	1.881–	1.280–2.778	0.001	2.158		1.245–3.741	0.006

**Table 4:** Predictors of CVD mortality in female, using competing risks models.

**Variables**	**Univariate analysis**			**Multivariate analysis**			
	HR	95%CI	*p* value	HR		95%CI	*p* value
Height	0.981	0.951–1.011	0.202	0.995		0.959–1.031	0.810
Depression (normal)	1.340	0.922–1.945	0.131	1.256		0.827–1.910	0.289
Smoking (no smoke)	0.827	0.534–1.278	0.390	–		–	–
Drink (no drink)	1.133	0.561–2.256	0.739	–		–	–
Sad event (experienced)	0.744	0.528–1.050	0.092	0.732		0.510–1.052	0.091
Exercise (always)	0.876	0.624–1.230	0.441	–		–	–
BADL (can take care)	1.020	0.442–2.371	0.962	–		–	–
IADL (can take care)	1.343	0.948–1.900	0.097	0.995		0.643–1.544	0.981
Marital status (have a spouse )	1.376	0.981–1.910	0.066	0.935		0.613–1.436	0.745
Assessment of health (normal)	1.211	0.821–1.823	0.331	–		–	–
Blood lipid (normal)	0.785	0.544–1.144	0.212	0.734		0.499–1.081	0.123
Diabetes (normal)	0.771	0.501–1.191	0.243	1.608		1.028–2.509	0.037
Age group (55–65)							
66–75	1.128	0.811–1.589	0.491	1.583		1.012–2.473	0.044
≥76	1.712	1.210–2.430	0.003	2.109		1.116–3.982	0.021
Blood-pressure(normal)							
Sbp > 120 or dbp > 80	0.616	0.335–1.133	0.120	0.962		0.477–1.941	0.911
Sbp ≥ 140 or dbp ≥ 90	1.520	1.051–2.190	0.027	1.739		1.102–2.740	0.018
Body mass index(normal)							
thin	1.273	0.859–1.871	0.230	1.072		0.697–1.652	0.750
overweight	0.651	0.373–1.132	0.130	0.704		0.392–1.263	0.241
obesity	0.668	0.379–1.181	0.161	0.674		0.371–1.219	0.189
Area(rural)							
suburban	0.761	0.509–1.140	0.193	0.636		0.366–1.115	0.112
urban	1.070	0.761–1.521	0.681	0.930		0.517–1.674	0.810
Diet(intermediate-type diets)							
sufficient nutrients	0.938	0.659–1.330	0.718	1.155		0.751–1.786	0.510
meat based diets	1.003	0.854–1.971	0.219	1.786		1.041–3.062	0.035

### 3.3. Fine and Gray Test

**Figure 3:**
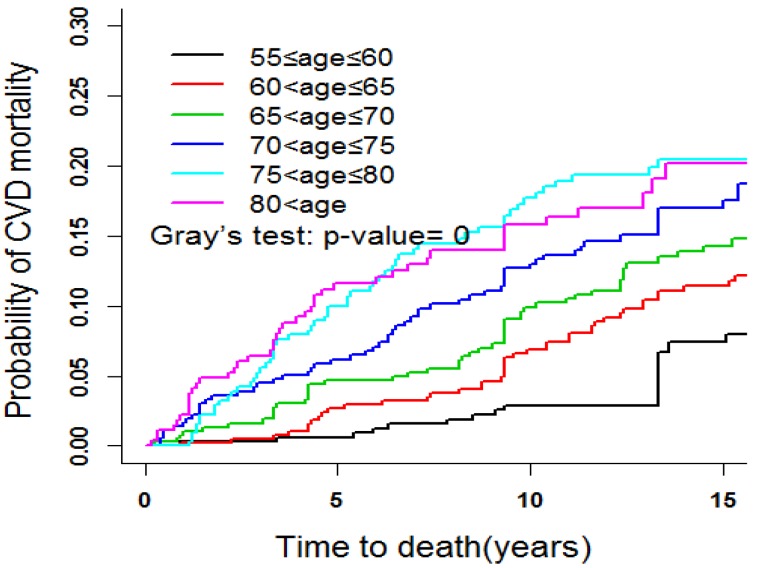
CIFs for death due to CVD: Compare the different age-groups.

**Figure 4:**
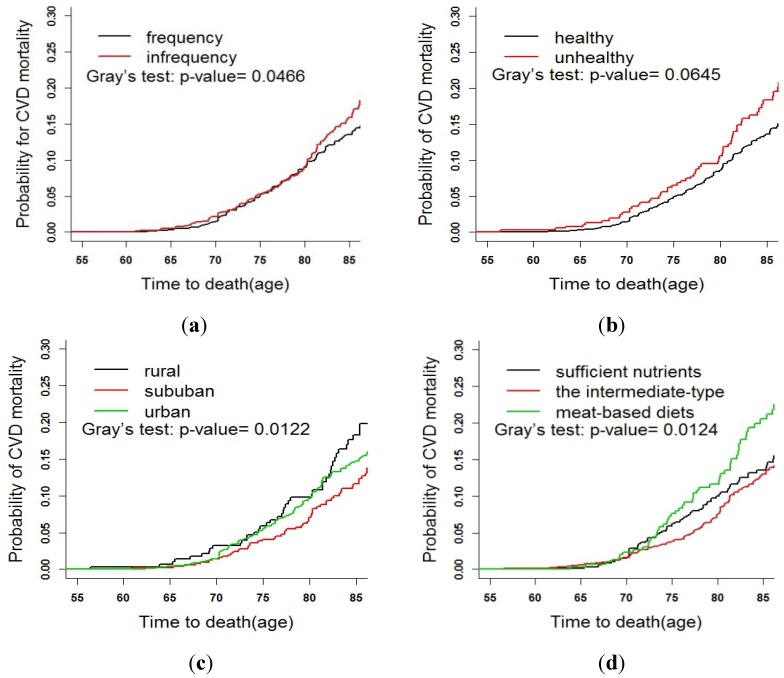
CIFs for death due to CVD: Comparing the different groups after adjusting age.

*Notes*: Abbreviations: (a) CIFs for the exercise group and exercise infrequently group; (b) CIFs for the results of self-health assessment; (c) CIFs for the different places of residence; (d) CIFs for the different diet habits groups.

In order to determine the tendency of CVD mortality in different age groups, Gray’s test was used to compare the CIFs for the six age groups (Figure 3). After five years from the beginning of the follow-up, the CIF of CVD mortality increased with the increasing age, and the elderly aged between 75 and 79 had the highest (*p* < 0.001). Gray’s test was also used to compare the CIFs of other groups, including gender, marital status, self-assessed health, depression, *etc.* Age of death was the abscissa to adjust the effect of age distribution in different groups. Several results are listed in Figure 4. As shown in Figure 4a, the mortality of the elderly who exercise infrequently was significantly higher than that of those who exercise frequently and regularly (*p =* 0.047), and that of subjects with poor self-assessed health were higher than that of those with good self-assessed health (*p =* 0.065) (Figure 4b). As shown in Figure 4c, the CVD mortalities among the different places of residence were statistically significant (*p =* 0.012). It was the highest for the elderly living in a rural area, the CIF at aged 85 of the rural area reached 0.23, the elderly in the suburban area came second and urban inhabitants had the lowest (CIF ≤ 0.15). For dietary intake, the mortality of subjects of meat-based diets was the highest, and that of the intermediate-type diets with balanced diet was the lowest (*p* = 0.012) (Figure 4d).

## 4. Discussion

Previous studies [32,33,34] have focused primarily on examining the effect of a single risk factor on mortality, rather than developing a more comprehensive model that incorporates the effects of multiple risk factors. Our findings confirm those in other studies, showing strong and significant associations of gender, age, blood pressure, residence place, and diet with the risk of CVD mortality in elderly people. Advanced age, high blood pressure, rural residence, and consuming more meat were associated with higher CVD mortality. Previous researches have confirmed that clinical manifestations and prognosis of CVD are likely altered in older people, because interactions that occur between age-associated cardiovascular changes in health and specific pathophysiologic mechanisms underlie a disease [35,36]. Our study also showed age was an independent risk factor associated with CVD morality after considering the competing risk events.

Age is by far the strongest determinant of CVD risk in the given multiple risk factors [37], and the prevalence of hypertension rises rapidly and steadily with aging. Hypertension might even lead to the further increases of CVD morbidity and mortality [38].

In addition, a healthier diet is associated with a lower risk of recurrent CVD events among people aged over 55 years with CVD [39]. A healthier diet indicates more frequent consumption vegetables and fruits as well as a higher consumption of fish relative to meat, poultry, and eggs [40]. In a multicenter trial in Spain, a Mediterranean diet supplemented with extra-virgin olive oil or nuts was confirmed to reduce the incidence of major cardiovascular events [41,42,43]. This research likewise showed more frequent intake of meat (the third group: Meat-based diets) increased the risk of death from CVD (HR = 1.578). The participants in this group consumed less of soy foods, fruits, vegetables, and fish, as well as more frequently consumed grains (≥350 grams per person per day), meat (>twice a week). Furthermore, the percentage of animal oils intake in this group was higher than in the other diet groups. According to our previous study [22], the elderly in the sufficient nutrients group primarily lived in the urban area and they always took breakfast and less meat, had more frequent consumption of soy foods, fruits, vegetables, and fish, whereas, the elderly in meat-based diet group primarily lived in the rural area. This study showed that the CVD mortality of residents in rural areas was significantly higher than those in urban and suburban ones (*p =* 0.012). Besides the dietary factor, it may be also related with the health systems in rural areas are not well equipped and medical resources are sparse.

Aside from that, evidence from the Framingham heart study has confirmed that cumulative long-term physical activity has a protective effect on the incidence of CVD-attributable mortality compared with the long-term physical inactivity [44]. This was consisted with our results. The result of Gray’s test showed that the cumulative CVD mortality of the elderly who exercised regularly were significantly lower than those without a long-term physical activity (*p =* 0.047), and a recent study in Canadians also confirms that the risk of CVD death decreases with the frequency of physical activity [45].

Additionally, our study confirmed that taller height may contribute to deceased CVD mortality risk (Table 3), which was consisted with a study in Chinese people in 2011 [46]. By Gray’s test, poor self-assessed health was markedly associated with higher CVD mortality (*p =* 0.065).

At a methodological standpoint, several advantages exist in using competing risks survival analysis to analyze the composite endpoint of mortality. Unlike the Cox proportional hazards regression model, in which risk factors are constrained to have common associations with all components of the outcome and ignoring the competing risks, this competing risk model allows for several risk factors to have different associations with single causes of death [47,48]. This study also has several limitations. Risk factors in the competing risk model were not updated year by year. The risk factor status at the beginning of the follow-up was used in order to avoid the possibility of any clinical or subclinical disease affecting the risk factor status.

## 5. Conclusions

At the end of follow-up, considering the competing risks, the CIF of CVD death was 0.19, CBVD was 0.17, and cancer was 0.10. A competing risk model was developed for a specific cause (CVD) of mortality in this study. Increasing age, raised blood pressure, living in a rural area and frequent consumption of meat was associated with higher CVD mortality of the elderly in Beijing, China. The study provided foundation of decision-making and instruction for the controlling and prevention of CVD. In the future, competing risk model could be used to identify individuals who are at higher risk of specific mortality.
